# Ouzo Effect Examined at the Nanoscale via Direct Observation of Droplet Nucleation and Morphology

**DOI:** 10.1021/acscentsci.2c01194

**Published:** 2023-03-08

**Authors:** Maria A. Vratsanos, Wangyang Xue, Nathan D. Rosenmann, Lauren D. Zarzar, Nathan C. Gianneschi

**Affiliations:** † Department of Materials Science & Engineering, Northwestern University, Evanston, Illinois 60208, United States; ‡ Department of Chemistry, The Pennsylvania State University, University Park, Pennsylvania 16802, United States; § Department of Materials Science and Engineering, The Pennsylvania State University, University Park, Pennsylvania 16802, United States; ∥ Materials Research Institute, The Pennsylvania State University, University Park, Pennsylvania 16802, United States; ⊥ International Institute for Nanotechnology, Simpson Querrey Institute, Chemistry of Life Processes Institute, Northwestern University, Evanston, Illinois 60208, United States; # Department of Chemistry, Department of Biomedical Engineering, Department of Pharmacology, Northwestern University, Evanston, Illinois 60208, United States

## Abstract

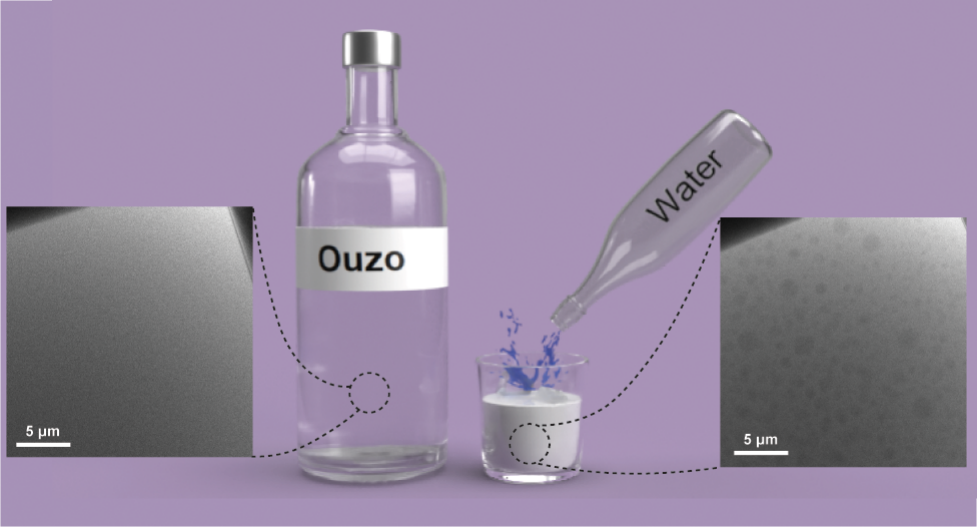

Herein, we present the direct observation via liquid-phase transmission electron microscopy (LPTEM) of the nucleation and growth pathways of structures formed by the so-called “ouzo effect”, which is a classic example of surfactant-free, spontaneous emulsification. Such liquid–liquid phase separation occurs in ternary systems with an appropriate cosolvent such that the addition of the third component extracts the cosolvent and makes the other component insoluble. Such droplets are homogeneously sized, stable, and require minimal energy to disperse compared to conventional emulsification methods. Thus, ouzo precipitation processes are an attractive, straightforward, and energy-efficient technique for preparing dispersions, especially those made on an industrial scale. While this process and the resulting emulsions have been studied by numerous indirect techniques (e.g., X-ray and light scattering), direct observation of such structures and their formation at the nanoscale has remained elusive. Here, we employed the nascent technique of LPTEM to simultaneously evaluate droplet growth and nanostructure. Observation of such emulsification and its rate dependence is a promising indication that similar LPTEM methodologies may be used to investigate emulsion formation and kinetics.

## Abstract 2

The Ouzo effect is a useful low energy emulsification strategy, which we here induced and observed in real time via *in situ* microscopy to better understand its mechanism.

## Introduction

**Figure 1:**
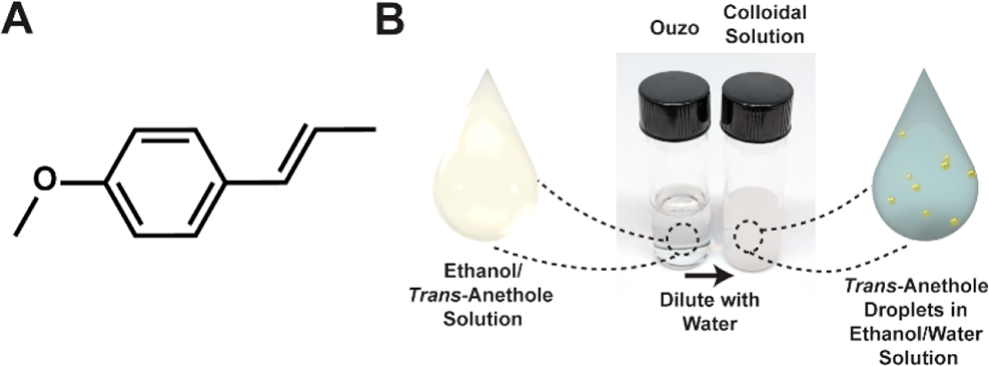
Structure of *trans*-anethole and depiction of the ouzo effect. (A) Structure of *trans*-anethole, the small molecule that results in the anise taste. (B) Photographs of the ethanol/*trans*-anethole solution before (left) and after (right) the addition of water, with schematic depictions shown as insets below.

The ouzo effect is a well-known phenomenon occurring in alcohols flavored with anise (including ouzo, arak, pastis, and raki) (Figure 1). The distinctive licorice flavoring of these beverages is the result of the anise extract, *trans-*anethole (melting point = 20 °C, Figure 1A).^1^ When these drinks (approximately 40% v/v ethanol in water and approximately 1% *trans-*anethole)^2^ are sufficiently diluted with water, they become opaque (Figure 1B). This opacity is the result of the precipitation of *trans-*anethole droplets, as the oil is insoluble in water.^1^ This effect is generalized for ternary systems, wherein the requirement is that one cosolvent (A) is soluble in two other solvents (B and C), but wherein B and C are immiscible with each other. Thus, when B is added to a mixture of A and C, it mixes with A and forces C to phase separate.^3^

Despite the ubiquity of this phenomenon, it has only recently attracted scientific attention, and was first named in 2003 in Vitale and Katz’s seminal work.^1^ Since that publication, numerous subsequent efforts to elucidate and understand the mechanism of ouzo droplet formation have emerged,^3−9^ and such low-energy emulsification strategies have found numerous applications from drug encapsulation to material templating.^8,10−17^ This is a very promising area of study, as the ouzo effect is a definitive example of spontaneous, surfactant-free emulsification, wherein minimal energy is required to disperse the insoluble phase and yet results in small, low dispersity, homogeneous droplets.^16,18^ Further, these droplets show astounding stability despite the system’s lack of surfactant stabilizers (the standard mechanism by which emulsion shelf life is extended), for reasons yet eluding researchers.^1^ Thus, the ouzo effect has the potential to allow the straightforward scale up of many emulsified products, as it is difficult to achieve sufficient shear on industrial scales, all without requiring the addition of surfactants, which may adversely affect formulations and are often environmentally detrimental.^19,20^

Despite the body of work in this area, a satisfactory understanding of the origin of this stability eludes researchers.^3^ Many recent efforts regarding the ouzo effect have focused on understanding the so-called “pre-ouzo” phase region, wherein weakly associated structures on the order a few nanometers are formed, prior to the evolution of the more stable droplets.^21−24^ While these structures are of interest, this size regime is limited to the initial time points of the effect, and will give little structure–property understanding of the metastable structures formed at later time points. These later structures are within the size regime that may be reliably resolved via electron microscopy techniques. Thus, we have chosen to focus on the behavior and growth of these droplets once in the metastable ouzo region.

In this work, we have not only been able to directly observe the nucleated *trans-*anethole droplets in their native state, but we have also been able to induce and observe said nucleation *in situ*. Such direct observation of emulsification via the ouzo effect has never been achieved before on the nanoscale and is only possible through liquid phase transmission electron microscopy (LPTEM) techniques. LPTEM is a nascent *in situ* microscopy technique which hermetically encapsulates picoliters of liquid sample against the vacuum environment of the microscope, allowing direct observation of solvated samples without fixation at unprecedented spatiotemporal resolutions.^25−46^ Notable advances in the understanding of nucleation and growth pathways,^33,43,47−52^ crystallization,^53−55^ nanoparticle behavior,^56−59^ self-assembly processes,^60−62^ thermoresponsive materials,^63,64^ and liquid–liquid phase separation^65−68^ have been achieved via LPTEM since its inception. One of the unique benefits of LPTEM for the study of liquid systems is that the contrast is directly proportional to the densities of the materials being studied. Other microscopy techniques, such as optical and super-resolution microscopy, are dependent on the refractive index of the materials or the inclusion of tags, respectively.^69^ Further, though super-resolution microscopy may be able to get comparable spatial resolution in some cases, such imaging is dependent on the inclusion of fluorescent dyes, which inherently raises uncertainty with respect to the identification and assignment of phases. By contrast, LPTEM yields contrast as a function of density differential, which allows the unambiguous assignment of phases as a function of intensity and contrast. Additionally, LPTEM is useful in its relative simplicity—image acquisition and processing are straightforward, and minimal postprocessing or algorithmic deconvolution is needed to interpret the data, which further permits improved temporal resolution. Here, we use this technique not only to study multiphase solvated systems, but to also introduce other solvents via microfluidic lines and ports built into commercial LPTEM holders (Figure S1). Studies of mixed phase systems via LPTEM, and specifically, the *in situ* mixing of multiple phases, remain an unexplored area of the field. Other works in this area have previously reported *in situ* observation of liquid–liquid phase separations, primarily in systems with amphiphilic block copolymers and intrinsically disordered proteins.^65,66,68,70^ Here, we use LPTEM to observe *in situ* emulsification events of small molecules such as *trans-*anethole. Further, this present work is the first *in situ* observation of so-called “surfactant-free microemulsions” (SFMEs). Observation of this process allows us to directly observe the morphology and evolution of emulsions produced via the ouzo effect.

## Results and Discussion

**Figure 2:**
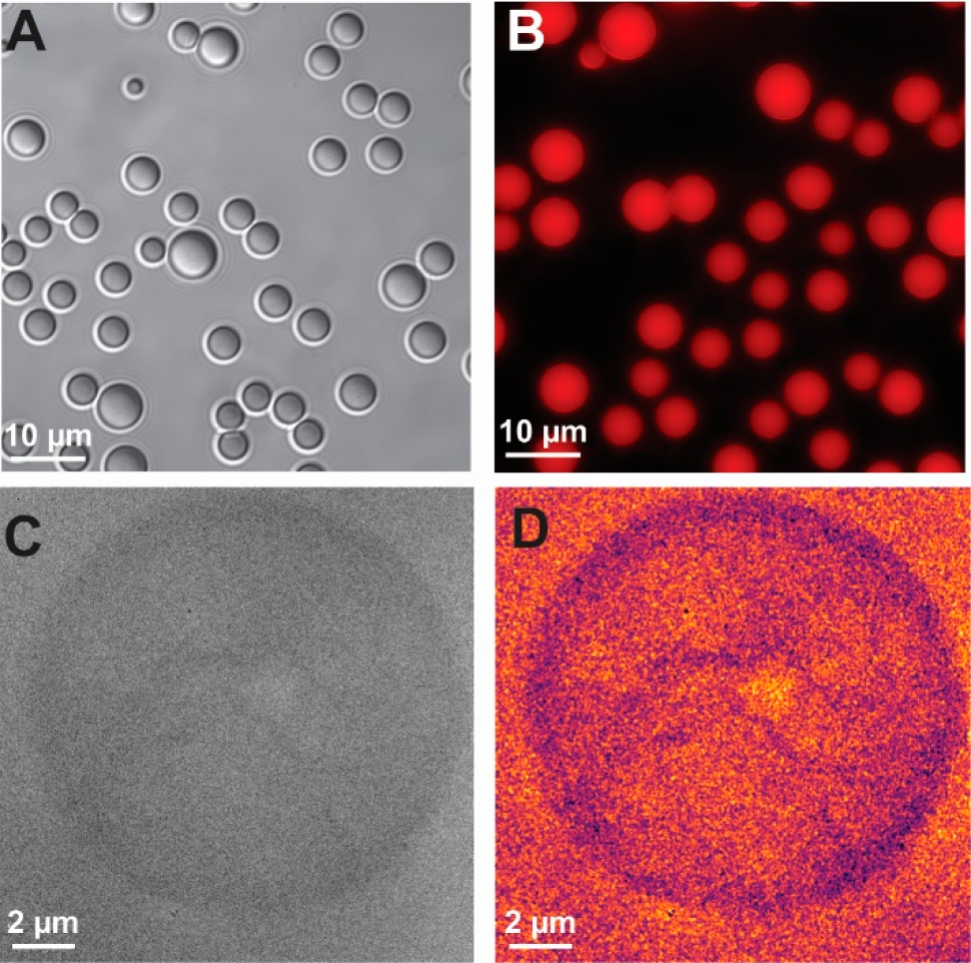
Multimodal microscopy of preformed *trans*-anethole droplets. (A) Brightfield optical and (B) fluorescence microscopy of bulk ouzo droplets. Droplets in (B) contained added Nile Red dye. (C) TEM micrograph of ouzo droplets formed in bulk from a 5 v% *trans*-anethole solution and loaded into the liquid cell. (D) False color image processing applied to TEM micrograph.

Based on previous successes imaging the morphology of traditional, surfactant-containing emulsions formed in the bulk,^71^ we started by imaging the preformed ouzo droplets to investigate whether they are of sufficient contrast to resolve *in situ* (Figure 2). Shown here with complementary optical and fluorescence microscopy (Figure 2A,B), we see that droplets of the same size regime are visible in the liquid cell experiments, compressed as it were, within the confines of the liquid cell (Figure 2C). The optical and fluorescence microscopy was carried out by adding drops of DI water to induce the ouzo effect in 20 v% *trans-*anethole solution, analogously to the LPTEM set up (Figure S2). We can conclude that the dark droplets in the LPTEM images are the *trans-*anethole-rich regions, given the higher density of this phase with respect to ethanol. Closer inspection of the TEM micrographs reveals internal structuring, which we have here used false color to emphasize (Figure 2D). Interestingly, internal structures of this type have not been previously seen in other investigated emulsion formulations. To further probe this structuring, we have also applied some basic image processing to aid visualization of the internal structure. This not only emphasizes the ringed structure of the droplets, but also reveals internal structuring as well.

**Figure 3:**
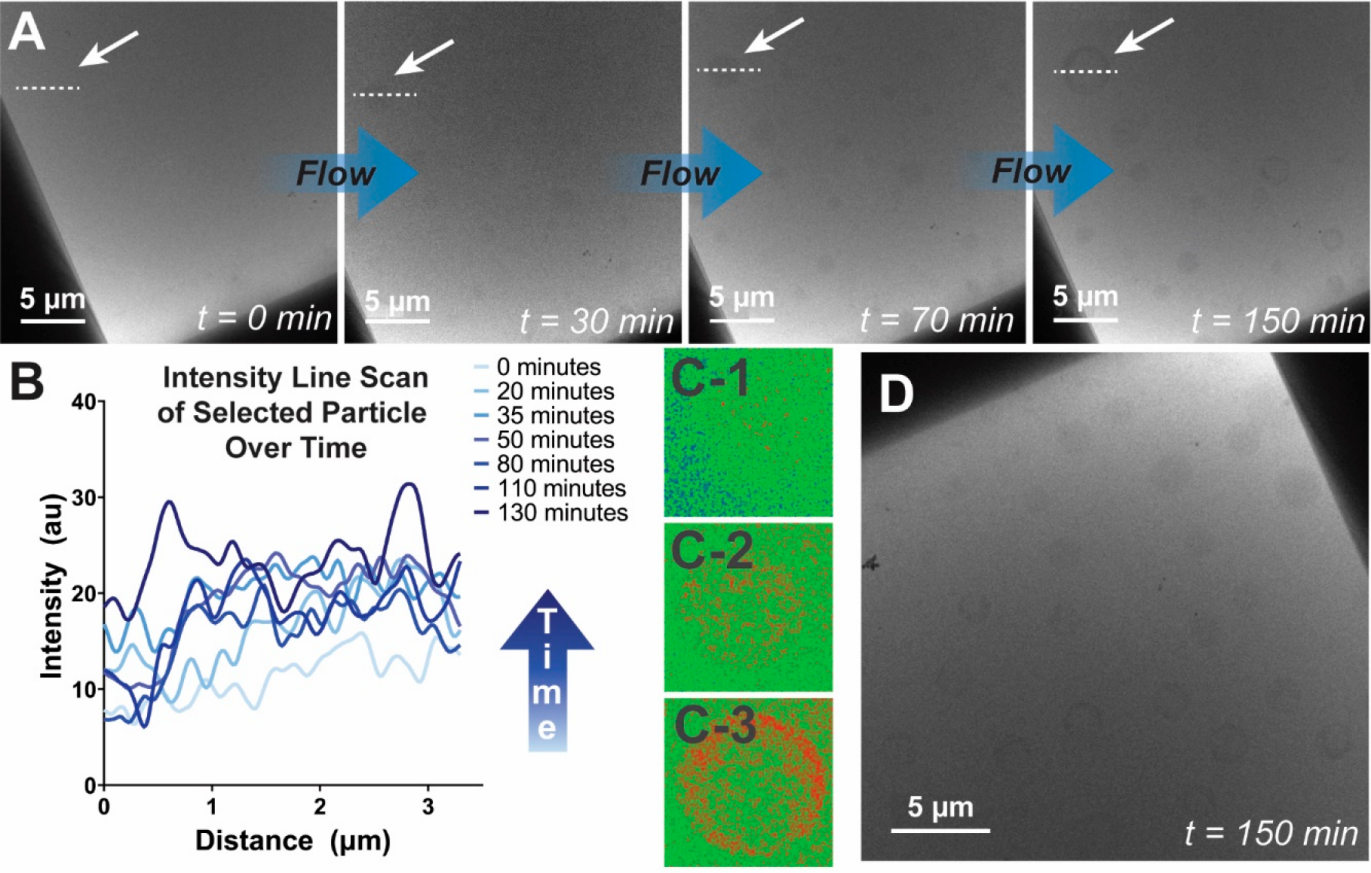
Time series of the ouzo effect in a solution of 20 v% *trans*-anethole in ethanol, diluted at a rate of 3 μL/min. (A) Initial images reveal no structures, and droplets appear, grow, and develop a ringed morphology during continued flow and stroboscopic imaging (to minimize fluence). Arrow indicates selected region for analysis. (B) Line scan across a representative droplet (denoted by white arrows and dashed lines) from the time series in A to demonstrate the change in pixel intensity over time. Greater intensity indicates darker pixels, corresponding to greater contrast in the image. Line scans were taken across the area indicated with the white arrow, and a representative line scan is depicted in the image at *t* = 150 min. (C1–3) Cropped image of analyzed droplet (as indicated with white arrows) with false coloration applied to enhance visibility of evolution in time (30, 70, and 150 min, respectively). (D) Micrograph of a less-imaged corner of the liquid cell at the end of the experiment (*t* = 150 min). These droplets demonstrate that their presence and structure does not rely on incident electron beam.

Given that the visibility of *trans-*anethole droplets had been established, we could investigate the formation of the phase separation by harnessing the microfluidic capabilities of the liquid cell holder, which allow us to flow solutions into the sample chamber during imaging. Initially, we studied a 20 v% *trans-*anethole solution diluted at a rate of 3 μL/min and were able to observe the formation and growth of oil droplets upon dilution of the ethanol solution (Figure 3). These droplets faintly appeared after approximately 30 min of dilution and exhibited growth and morphological evolution under continued stroboscopic imaging (Figure 3A). To ensure that a true time zero image was captured, the flow lines were left empty of diluent to prevent premature mixing, and the flow of water was not started until representative images of the sample as loaded had been taken. Some simple calculations considering the geometry of the microfluidic system and the relevant flow rates estimate that the water should enter the sample chamber between 15 and 40 min after initiation (see Supporting Information). Then, when the exterior volume is filled, diffusive mixing between this external reservoir and the narrow region of available sample surface area occurs. Thus, the observed nucleation of *trans*-anethole droplets at the 30 min mark is consistent with expectation.

These droplets are first visible at sizes over a micron, and much of the evolution in time beyond this point was in intensity, rather than size (Figure 3B), which results in a dark ring around the exterior of the droplet and a lighter interior (Figure 3C). Recent work has revealed the presence of 1 and 100 nm structures in the monophasic region, which are likely of insufficient contrast for visualization. Thus, we are most likely observing the larger structures resulting from phase separation.^72^ Unlike efforts to emulsify substances *in situ* via surfactants (Figure S3), these droplets appeared homogeneously, developing simultaneously across all visible areas (Figure 3D), indicating that the solvent had reached some critical concentration of water to render the *trans-*anethol insoluble. The presence of structuring in both these phase separated states and the preformed droplets may suggest that they undergo some internal microphase separation after nucleation. The changes in contrast suggest that an ethanol/water-rich region develops at the center of the droplet, while the shell remains predominantly composed of *trans*-anethole (as indicated by the relative contrasts).

**Figure 4:**
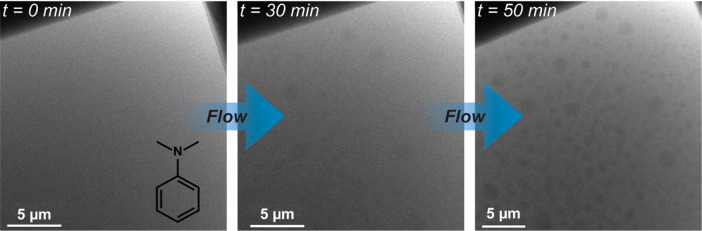
Nucleation of *N,N*-dimethylaniline droplets from 20 v% ethanol solution by dilution with water at 3 μL/min. Micrographs show progression from initial cell without structures to the appearance of high contrast oil droplets under continual dilution. Structure of *N,N*-dimethylaniline is shown as an inset in first panel.

Previously, the e-beam has been demonstrated to initiate polymerization under LPTEM imaging.^61^ To avoid the possibility such e-beam induced polymerization may cause these observations (given the unsaturated alkene present in *trans-*anethole), we additionally studied *N*,*N*-dimethylaniline as the oil phase. *N*,*N*-Dimethylaniline is also know to undergo the ouzo effect, but lacks unsaturated carbons, so any observed droplet formation cannot be the result of beam-induced polymerziation.^1^ Following an identical experimental protocol, (20 v% oil in ethanol by volume, 3 μL/min dilution), spontaneous nucleation was observed again, confirming that particle appearance is not the result of polymerization (Figure 4). Internal anisotropy of the structures was again observed, though in a manner less pronounced than for *trans-*anethole.

**Figure 5:**
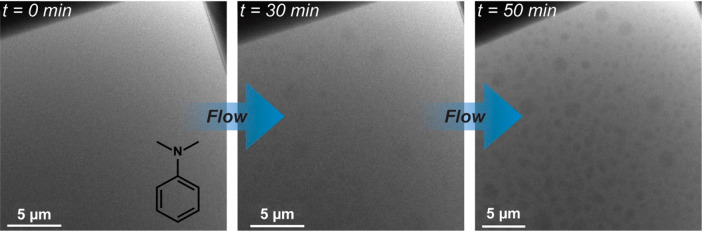
*In situ* formation and growth of confined *trans-*anethole droplets from a 5 v% *trans*-anethole in ethanol solution, diluted at a rate of 3 μL/min, and subsequent analysis. (A) Selected micrographs from a time series documenting the evolution of 3 droplets, denoted by arrows. (B) Plot of droplet diameter growth in time and accompanying logistic fits of data. Logistic fitting here indicates the growth mechanism does not follow typical ripening rates. (C) μFTIR spectra of experimental SiN_*x*_ chip (pictured in inset). Spectra were acquired at the three indicated locations, Spectrum 2 being the imaged region, and Spectra 1 and 3 being outside the imaging region. Black spectrum shows unimaged control chip, with dropcast *trans-*anethole solution for reference. Here, we see the spectra match closely between the imaged and unimaged regions, indicating that minimal damage has occurred to the material.

To quantitatively characterize the observed particle formation processes, growth rates were measured and a post-mortem micro-Fourier Transform Infrared Spectroscopy (μFTIR) experiment was performed (Figure 5). Sizes over time (Figure 5A) were manually measured and plotted as a function of time, with logistic fits applied to establish a growth rate (Figure 5B). To further investigate whether or not the active small molecule (*trans-*anethole) chemically degraded during observation, we used μFTIR analysis, which allowed for FTIR spectra to be taken of the imaged region on the micron scale and compared to spectra of the unimaged region and controls (Figure 5C). Here, the three spectra were acquired at the points indicated by arrows to generate two spectra of unimaged region (Scans 1 and 3) for comparison with the spectrum of imaged region (Scan 2). Additionally, a control sample was created by drop-casting the same solution on an unused SiN_*x*_ chip, which remained unimaged. Given that the signal from the imaged region matches the spectra of both the unimaged regions on the experimental chip and unimaged control, we are confident that the *trans-*anethole remains undamaged by the beam at these conditions (0.1 e^–^/Å^2^ s), and thus growth and evolution of the phase separated state is not the result of e-beam induced damage. Conversely, high-flux experiments (>1 e^–^/Å^2^ s) showed large discrepancies between Scan 2 and Scans 1 and 3, indicating that this post-mortem technique can successfully differentiate between intact and damaged material (Figure S4). Thus, we are sufficiently confident that the observed growth is not the result of e-beam mediated damage and can draw conclusions from our evaluations of kinetics.

To probe the effect of sample composition and flow rate on growth rates, the aforementioned measurement protocol was followed for all data acquired, and complete data sets are available in the SI (Table S1). Growth kinetics were better fit to logistic models, rather than linear (Table S2). The *trans-*anethole concentration is directly correlated with the number of nucleation events observed in the cell, consistent with findings in the literature (Figure S6).^4^ Several mechanisms of formation and growth for ouzo emulsions have been hypothesized, and these follow the destabilization methods of classical emulsions: diffusion-driven processes (ripening), or combination events (coalescence).^9,12^ Here, no coalescence events were observed under any conditions, despite previously establishing that such events are visible by this technique at comparable time and length scales^71^ However, droplet growth was consistently logistic in time, which is contrary to classical ripening mechanisms, wherein *r*^3^ is linear in time.^73^ Thus, our findings strongly suggest that the predominant mechanism here is the diffusion-driven growth of initial nuclei, but not Ostwald ripening. We have previously considered the effect of spatial constraints on droplets in this size regime. Indeed, droplets with diameters on the micron scale exist as spheroids *in situ*, and have different curvature and surface areas than spheres typically considered in the bulk.^71^ However, these deviations act in an approximately equal and opposite manner so as to yield an effective Ostwald ripening rate of the same order of magnitude as predicted. Thus, we do not consider it is likely that the anomalous ripening rate results from *in situ* artifacts. It is also possible that coalescence events may occur while the sample is in the preouzo region, which would suggest that it occurs on such small length scales that we are unable to resolve them.^22^ If this is the case, the coalescence events have concluded prior to the resolution of the confined droplets observed via LPTEM. Our findings support the theory that such droplets grow via diffusion-driven mechanisms, or that such growth happens via a two-step process, the second part of which we are observing.^71^

To determine if nucleation behavior changes with *trans*-anethole concentration, formulations ranging from 5 to 20 v% *trans-*anethole in ethanol were examined, which were diluted with deionized water at rates from 1 to 3 μL/min (Figures S5–S7, Table S1). With such models, we can compare growth constants, *k*, to evaluate differences in conditions. A weak concentration dependence was observed, but not determined to be statistically significant.^4^ Flow rate was shown to have a significant impact on growth rate at both the 20% and 10% *trans-*anethole conditions. Nucleation at slower flow rates had significantly lower growth rates than those nucleated at higher flow rates, (Figure S7).^4^ This supports the hypothesis that a more rapid addition of water would result in the nucleation of fewer, larger droplets as a result of kinetic trapping. It is possible that these variations were altered by the confinement of the liquid cell, which significantly limits diffusion and constrains particle behavior to the *x*–*y* plane.^42,74,75^ Thus, we are hesitant to draw quantitative conclusions from these rate evaluations, and we consider the significance to be the observed trends between flow rates and compositions, as well as the structural observations made.

**Figure 6:**
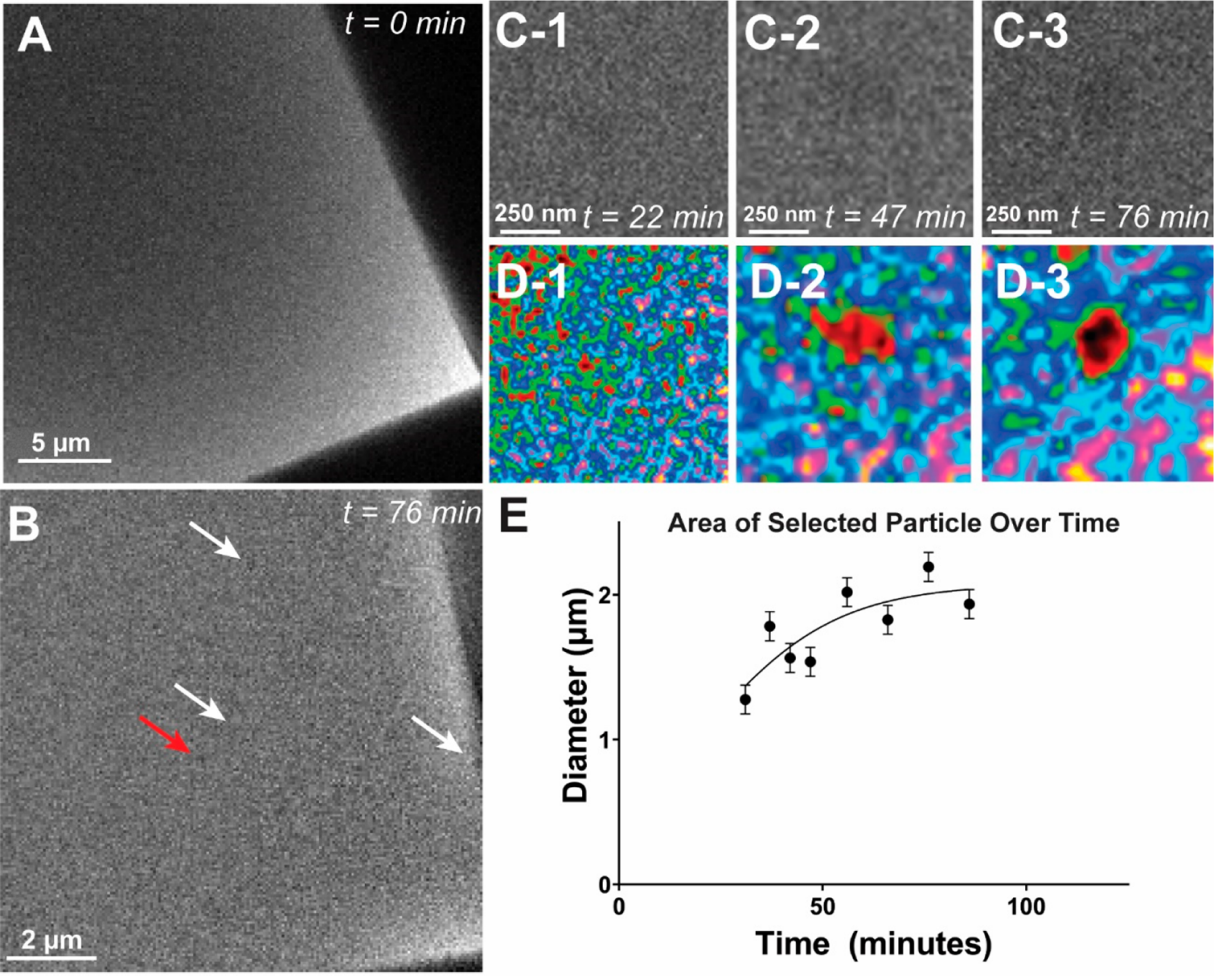
Nucleation in a commercial ouzo sample. (A) Initial image of commercial ouzo, no structures visible. (B) Micrograph of ouzo after dilution with water at 5 μL/min for 76 min, with droplets indicated by arrows. Red arrow denotes droplet for subsequent analysis. (C1–C3) Sequential micrographs of droplet indicated by red arrow. Micrographs have been cropped to a region of interest around the droplet and have been background subtracted for visibility. (D1–D3) False coloration applied to above micrographs to enhance visibility of droplet nucleation and growth. (E) Plot of droplet area growth in time for selected region indicated above.

In the interest of authenticity, experiments were also carried out with a commercial ouzo formulation. 12 Ouzo was loaded into the liquid cell and diluted at a rate of 3 μL/min (Figure 6). Though the exact *trans-*anethole content in this formulation is unknown, it is reasonable to assume that it is around 1 v%, and we assumed that the overall solvent composition is 42 v% ethanol, with the remainder being water.^2^ Here, we again saw the evolution of the phase separation after an initial absence of structures at approximately 30 min, though these structures were much smaller than those seen previously using pure *trans*-anethole (Figure 6A). By following our established image processing protocol, we were able to visualize growth and densification in time (Figure 6B). We can use this data to quantify droplet growth by evaluating the area above a given background intensity, which shows a logistic growth (Figure 6C).

“Ringing” was observed across the majority of experiments indicating microphase separation of an ethanol-rich region within the *trans-*anethole droplet (particularly evident in Figure 2C and D and Figure 3A and C). We were unable to observe this microphase separation inside droplets with optical or fluorescence microscopy, and it has not been reported under any other conditions or compositions.^71^ This separation may be the result of local concentration gradients appearing around the droplet and inducing further structuring as a result of preferential substrate wetting. Thus, while this may be an artifact of the confined *in situ* flow cell environment, it brings up interesting possibilities and opportunities to intentionally generate such structures via designed nanoconfinement and surface chemistry. Similar events have been observed on other length scales.^72^

It is notable that coalescence was not observed for this system of emulsions, as it lends credence to hypotheses that ripening-type behaviors dominate the evolution of such emulsions in time.^72,76^ Further, as is the nature of microscopy, quantitative evaluation can be made from direct observations as compared to the suite of parallel techniques which must otherwise be employed to glean similar information.^76^

## Conclusions

The observation of both the formation and morphology of spontaneously emulsified oil in water droplets via the ouzo effect was achieved *via in situ* liquid phase transmission electron microscopy. Here, mechanistic studies of droplet formation and growth found that both the growth constant and number of nucleated, confined droplets was directly proportional to *trans*-anethole concentrations. Coalescence was not observed, and kinetics of observed ripening did not match established models of Ostwald ripening, perhaps suggesting a more rapid depletion of smaller species. Interestingly, internal structuring was observed *in situ* but not by correlative fluorescence microscopy. Post-mortem characterization and strategic controls demonstrate that these morphologies and growth are not the result of e-beam induced processes and may be the result of surface interactions triggered by confinement. Such insights are not possible by indirect characterization methods commonly used and lend credence to some of the theorized mechanisms of stability. More importantly, it is increasingly clear from this study and from others in the field that LPTEM can provide a complementary tool in the examination of soft matter and solvated systems undergoing dynamic processes and changes of state. Indeed, given the demonstrated successful imaging of this relatively simple liquid–liquid phase separation system, we anticipate that this *in situ* microscopy technique may be useful in gaining insight into other emulsifications, such as phase inversion temperature, and more complex multiphase systems.

## Methods

A brief overview of methods is provided here. More details materials and methods information can be found in the Supporting Information.

### LPTEM Sample Preparation

Liquid cells were prepared as previously described in the literature.^71^ Briefly, solutions of ethanol and *trans-*anethole were drop cast onto nonglow discharged SiN_*x*_ chips in amounts less than 0.8 μL. Top chips were deposited such that the windows were aligned orthogonally, and the holder was sealed with the top clamp assembly. Lines were left unfilled with solvent so as to avoid premature dilution of the sample with diluent. The holder was then pumped down using the external pumping station, and the cell windows were visually inspected using the attached optical microscope. Once the cell has reached 8.6 × 10^–6^ mbar, the fluidic ports were unsealed and the flow line was attached, so as to ensure cell integrity during dilution. This flow line was attached to a syringe and syringe pump, which were used to flow in water to the cell at rates from 1 to 5 μL/min. A JEM-ARM300F transmission electron microscope operating at a voltage of 300 keV and current of 15 μA (FEG source) was used for liquid cell experiments. Images were acquired via Gatan 2*k* × 2*k* OneView IS CMOS camera via Gatan Digital Micrograph imaging software with exposures of 1 s.

### Image Analysis

Images were binned to a resolution of 1*k* × 1*k* and background adjusted by subtracting a heavily Gaussian blurred copy of the same. This helps to compensate for the gradient background present from the bulged liquid layer, and doing so helps to enhance visibility of structures. Structures were then manually measured in ImageJ and resulting data were analyzed in GraphPad Prism.

### MicroFTIR

A Bruker MicroFTIR was used to analyze the windows of the SiN_*x*_ chips after the conclusion of the experiment as a post-mortem characterization to confirm the molecular integrity of our small molecules. Given the thickness of the SiN_*x*_ chips and windows, it was necessary to run these experiments in reflectance mode, which also required 500 scans in order to generate signal with sufficient intensity. Two cm wavelength resolution was used.

## Supporting Information Available

Material and experimental details including sample preparation and imaging conditions, supporting Figures S1–S7, Tables S1–S2, supplemental droplet growth data and discussion (PDF)

## References

[ref1] Vitale S. A.; Katz J. L. Liquid Droplet Dispersions Formed by Homogeneous Liquid-Liquid Nucleation: “The Ouzo Effect.. Langmuir 2003, 19 (10), 4105–4110. 10.1021/la026842o.

[ref2] Vendramin V.; Pesce A.; Vincenzi S. Anethole Stability in Aniseed Spirits: Storage Condition Repercussions on Commercial Products. Beverages 2021, 7 (4), 73 10.3390/beverages7040073.

[ref3] Goubault C.; Iglicki D.; Swain R. A.; McVey B. F. P.; Lefeuvre B.; Rault L.; Nayral C.; Delpech F.; Kahn M. L.; Chevance S.; Gauffre F. Effect of Nanoparticles on Spontaneous Ouzo Emulsification. J. Colloid Interface Sci. 2021, 603, 572–581. 10.1016/j.jcis.2021.06.104. 34216953

[ref4] Sitnikova N. L.; Sprik R.; Wegdam G.; Eiser E. Spontaneously Formed Trans-Anethol/Water/Alcohol Emulsions: Mechanism of Formation and Stability. Langmuir 2005, 21 (16), 7083–7089. 10.1021/la046816l. 16042427

[ref5] Prévost S.; Krickl S.; Marčelja S.; Kunz W.; Zemb T.; Grillo I. Spontaneous Ouzo Emulsions Coexist with Pre-Ouzo Ultraflexible Microemulsions. Langmuir 2021, 37 (13), 3817–3827. 10.1021/acs.langmuir.0c02935. 33724851

[ref6] Lu Z.; Schaarsberg M. H. K.; Zhu X.; Yeo L. Y.; Lohse D.; Zhang X. Universal Nanodroplet Branches from Confining the Ouzo Effect. Proc. Natl. Acad. Sci. U. S. A. 2017, 114 (39), 10332–10337. 10.1073/pnas.1704727114. 28894002 PMC5625903

[ref7] Botet R. The “Ouzo Effect”, Recent Developments and Application to Therapeutic Drug Carrying. J. Phys. Conf Ser. 2012, 352 (1), 012047 10.1088/1742-6596/352/1/012047.

[ref8] Goubault C.; Sciortino F.; Mongin O.; Jarry U.; Bostoën M.; Jakobczyk H.; Burel A.; Dutertre S.; Troadec M. B.; Kahn M. L.; Chevance S.; Gauffre F. The Ouzo Effect: A Tool to Elaborate High-Payload Nanocapsules. J. Controlled Release 2020, 324, 430–439. 10.1016/j.jconrel.2020.05.023. 32439361

[ref9] Iglicki D.; Goubault C.; Nour Mahamoud M.; Chevance S.; Gauffre F. Shedding Light on the Formation and Stability of Mesostructures in Ternary “Ouzo” Mixtures. J. Colloid Interface Sci. 2023, 633, 72–81. 10.1016/j.jcis.2022.11.060. 36436349

[ref10] Kempe H.; Kempe M. Ouzo Polymerization: A Bottom-up Green Synthesis of Polymer Nanoparticles by Free-Radical Polymerization of Monomers Spontaneously Nucleated by the Ouzo Effect; Application to Molecular Imprinting. J. Colloid Interface Sci. 2022, 616, 560–570. 10.1016/j.jcis.2022.02.035. 35228051

[ref11] Lamb A.; He F.; Zhai S.; Zhao H. Silk Fibroin Supraparticles Created by the Evaporation of Colloidal Ouzo Droplets. AIP Adv. 2021, 11 (8), 085125 10.1063/5.0057228.

[ref12] Aschenbrenner E.; Bley K.; Koynov K.; Makowski M.; Kappl M.; Landfester K.; Weiss C. K. Using the Polymeric Ouzo Effect for the Preparation of Polysaccharide-Based Nanoparticles. Langmuir 2013, 29 (28), 8845–8855. 10.1021/la4017867. 23777243

[ref13] Wang Y.; Zeng B.; Zhao Y.; Li S.; Zhang X. Formation of Polystyrene Microlenses via Transient Droplets from the Ouzo Effect for Enhanced Optical Imaging. J. Phys. Chem. C 2019, 123 (23), 14327–14337. 10.1021/acs.jpcc.9b00587.

[ref14] Peng S.; Xu C.; Hughes T. C.; Zhang X. From Nanodroplets by the Ouzo Effect to Interfacial Nanolenses. Langmuir 2014, 30 (41), 12270–12277. 10.1021/la502821m. 25262570

[ref15] Lepeltier E.; Bourgaux C.; Couvreur P. Nanoprecipitation and the “Ouzo Effect”: Application to Drug Delivery Devices. Adv. Drug Deliv Rev. 2014, 71, 86–97. 10.1016/j.addr.2013.12.009. 24384372

[ref16] Ganachaud F.; Katz J. L. Nanoparticles and Nanocapsules Created Using the Ouzo Effect: Spontaneous Emulsification as an Alternative to Ultrasonic and High-Shear Devices. ChemPhysChem 2005, 6 (2), 209–216. 10.1002/cphc.200400527. 15751338

[ref17] Cholakova D.; Vinarov Z.; Tcholakova S.; Denkov N. D. Self-Emulsification in Chemical and Pharmaceutical Technologies. Curr. Opin. Colloid Interface Sci. 2022, 59, 101576 10.1016/j.cocis.2022.101576.

[ref18] Klossek M. L.; Touraud D.; Zemb T.; Kunz W. Structure and Solubility in Surfactant-Free Microemulsions. ChemPhysChem 2012, 13 (18), 4116–4119. 10.1002/cphc.201200667. 23233274

[ref19] Surfactants in Consumer Products: Theory, Technology, and Application, Falbe J., Ed.; Springer Berlin Heidelberg: Berlin, Heidelberg, 1987. 10.1007/978-3-642-71545-7.

[ref20] McClements D. J.; Jafari S. M. Improving Emulsion Formation, Stability and Performance Using Mixed Emulsifiers: A Review. Adv. Colloid Interface Sci. 2018, 251, 55–79. 10.1016/j.cis.2017.12.001. 29248154

[ref21] Prévost S.; Krickl S.; Marčelja S.; Kunz W.; Zemb T.; Grillo I. Spontaneous Ouzo Emulsions Coexist with Pre-Ouzo Ultraflexible Microemulsions. Langmuir 2021, 37 (13), 3817–3827. 10.1021/acs.langmuir.0c02935. 33724851

[ref22] Marcus J.; Touraud D.; Prévost S.; Diat O.; Zemb T.; Kunz W. Influence of Additives on the Structure of Surfactant-Free Microemulsions. Phys. Chem. Chem. Phys. 2015, 17 (48), 32528 10.1039/C5CP06364G. 26593697

[ref23] Carteau D.; Pianet I.; Brunerie P.; Guillemat B.; Bassani D. M. Probing the Initial Events in the Spontaneous Emulsification of Trans-Anethole Using Dynamic NMR Spectroscopy. Langmuir 2007, 23 (7), 3561–3565. 10.1021/la062339q. 17315890

[ref24] Carteau D.; Bassani D.; Pianet I. The “Ouzo Effect”: Following the Spontaneous Emulsification of Trans-Anethole in Water by NMR. Comptes Rendus Chimie 2008, 11 (4–5), 493–498. 10.1016/j.crci.2007.11.003.

[ref25] Korpanty J.; Parent L. R.; Gianneschi N. C. Enhancing and Mitigating Radiolytic Damage to Soft Matter in Aqueous Phase Liquid-Cell Transmission Electron Microscopy in the Presence of Gold Nanoparticle Sensitizers or Isopropanol Scavengers. Nano Lett. 2021, 21, 1141–1149. 10.1021/acs.nanolett.0c04636. 33448858

[ref26] Patterson J. P.; Proetto M. T.; Gianneschi N. C. Soft Nanomaterials Analysed by in Situ Liquid TEM: Towards High Resolution Characterisation of Nanoparticles in Motion. Perspect Sci. (Neth) 2015, 6, 106 10.1016/j.pisc.2015.10.003.

[ref27] Parent L. R.; Gnanasekaran K.; Korpanty J.; Gianneschi N. C. 100th Anniversary of Macromolecular Science Viewpoint: Polymeric Materials by in Situ Liquid-Phase Transmission Electron Microscopy. ACS Macro Lett. 2021, 10 (1), 14–38. 10.1021/acsmacrolett.0c00595. 35548998

[ref28] Korpanty J.; Gnanasekaran K.; Venkatramani C.; Zang N.; Gianneschi N. C. Organic Solution-Phase Transmission Electron Microscopy of Copolymer Nanoassembly Morphology and Dynamics. Cell Rep. Phys. Sci. 2022, 3 (3), 100772 10.1016/j.xcrp.2022.100772.

[ref29] Parent L. R.; Vratsanos M.; Jin B.; de Yoreo J. J.; Gianneschi N. C. Chemical and Physical Transformations of Carbon-Based Nanomaterials Observed by Liquid Phase Transmission Electron Microscopy. MRS Bulletin 2020, 45, 727–737. 10.1557/mrs.2020.224.

[ref30] de Jonge N.; Ross F. M. Electron Microscopy of Specimens in Liquid. Nature Nanotechnology 2011, 6, 695–704. 10.1038/nnano.2011.161. 22020120

[ref31] Mirsaidov U. M.; Zheng H.; Casana Y.; Matsudaira P. Imaging Protein Structure in Water at 2.7 Nm Resolution by Transmission Electron Microscopy. Biophys. J. 2012, 102 (4), L15–L17. 10.1016/j.bpj.2012.01.009. 22385868 PMC3283772

[ref32] Wu H.; Friedrich H.; Patterson J. P.; Sommerdijk N. A. J. M. J. M.; Jonge N. Liquid-Phase Electron Microscopy for Soft Matter Science and Biology. Adv. Mater. 2020, 32 (25), 2001582 10.1002/adma.202001582. 32419161

[ref33] Woehl T. J.; Evans J. E.; Arslan I.; Ristenpart W. D.; Browning N. D. Direct in Situ Determination of the Mechanisms Controlling Nanoparticle Nucleation and Growth. ACS Nano 2012, 6 (10), 8599–8610. 10.1021/nn303371y. 22957797 PMC3482139

[ref34] Pu S.; Gong C.; Robertson A. W. Liquid cell transmission electron microscopy and its applications. R. Soc. open sci. 2020, 7, 191204 10.1098/rsos.191204. 32218950 PMC7029903

[ref35] Saarbrücken P. K. Liquid-Phase Electron Microscopy: Toward Direct Imaging of Self-Assembly Processes in Low-Atomic Number Colloidal Suspensions; 2021.

[ref36] Marchello G.; de Pace C.; Acosta-Gutierrez S.; Lopez-Vazquez C.; Wilkinson N.; Gervasio F. L.; Ruiz-Perez L.; Battaglia G.; Building C. I. 4D Imaging of Soft Matter in Liquid Water. bioRxiv 2021, 2021.01.21.427613 10.1101/2021.01.21.427613.

[ref37] Bae Y.; Ha M. Y.; Bang K. T.; Yang S.; Kang S. Y.; Kim J.; Sung J.; Kang S.; Kang D.; Lee W. B.; Choi T. L.; Park J. Conformation Dynamics of Single Polymer Strands in Solution. Adv. Mater. 2022, 34 (32), 2202353 10.1002/adma.202202353. 35725274

[ref38] Moreno-Hernandez I. A.; Crook M. F.; Jamali V.; Alivisatos A. P. Recent Advances in the Study of Colloidal Nanocrystals Enabled by in Situ Liquid-Phase Transmission Electron Microscopy. MRS Bull. 2022, 47 (3), 305–313. 10.1557/s43577-022-00287-5.

[ref39] Sung J.; Bae Y.; Park H.; Kang S.; Choi B. K.; Kim J.; Park J. Liquid-Phase Transmission Electron Microscopy for Reliable In Situ Imaging of Nanomaterials. Annu. Rev. Chem. Biomol. Eng. 2022, 13, 167–191. 10.1146/annurev-chembioeng-092120-034534. 35700529

[ref40] Kröger R.; Verch A. Liquid Cell Transmission Electron Microscopy and the Impact of Confinement on the Precipitation from Supersaturated Solutions. Minerals 2018, 8 (1), 21 10.3390/min8010021.

[ref41] Piffoux M.; Ahmad N.; Nelayah J.; Wilhelm C.; Silva A.; Gazeau F.; Alloyeau D. Monitoring the Dynamics of Cell-Derived Extracellular Vesicles at the Nanoscale by Liquid-Cell Transmission Electron Microscopy. Nanoscale 2018, 10 (3), 1234–1244. 10.1039/C7NR07576F. 29292437

[ref42] Yesibolati M. N.; Mortensen K. I.; Sun H.; Brostrøm A.; Tidemand-Lichtenberg S.; Mølhave K. Unhindered Brownian Motion of Individual Nanoparticles in Liquid-Phase Scanning Transmission Electron Microscopy. Nano Lett. 2020, 20 (10), 7108–7115. 10.1021/acs.nanolett.0c02352. 32678608

[ref43] Evans J. E.; Jungjohann K. L.; Browning N. D.; Arslan I. Controlled Growth of Nanoparticles from Solution with in Situ Liquid Transmission Electron Microscopy. Nano Lett. 2011, 11 (7), 2809–2813. 10.1021/nl201166k. 21619024 PMC3162246

[ref44] Chee S. W.; Baraissov Z.; Loh N. D.; Matsudaira P. T.; Mirsaidov U. Desorption-Mediated Motion of Nanoparticles at the Liquid-Solid Interface. J. Phys. Chem. C 2016, 120 (36), 20462–20470. 10.1021/acs.jpcc.6b07983.

[ref45] Ring E. A.; de Jonge N. Video-Frequency Scanning Transmission Electron Microscopy of Moving Gold Nanoparticles in Liquid. Micron 2012, 43 (11), 1078–1084. 10.1016/j.micron.2012.01.010. 22386765

[ref46] Abellan P.; Woehl T. J. Liquid Cell Electron Microscopy for the Study of Growth Dynamics of Nanomaterials and Structure of Soft Matter. In In-situ Characterization Techniques for Nanomaterials; Springer-Verlag Berlin Heidelberg, 2018; pp 1–31. 10.1007/978-3-662-56322-9_1.

[ref47] van Vleet M. J.; Weng T.; Li X.; Schmidt J. R. In Situ, Time-Resolved, and Mechanistic Studies of Metal-Organic Framework Nucleation and Growth. Chem. Rev. 2018, 118, 3681–3721. 10.1021/acs.chemrev.7b00582. 29514005

[ref48] Wang M.; Park C.; Woehl T. J. Quantifying the Nucleation and Growth Kinetics of Electron Beam Nanochemistry with Liquid Cell Scanning Transmission Electron Microscopy. Chem. Mater. 2018, 30 (21), 7727–7736. 10.1021/acs.chemmater.8b03050.

[ref49] Radisic A.; Ross F. M.; Searson P. C. In Situ Study of the Growth Kinetics of Individual Island Electrodeposition of Copper. J. Phys. Chem. B 2006, 110 (15), 7862–7868. 10.1021/jp057549a. 16610883

[ref50] Vailonis K. M.; Gnanasekaran K.; Powers X. B.; Gianneschi N. C.; Jenkins D. M. Elucidating the Growth of Metal-Organic Nanotubes Combining Isoreticular Synthesis with Liquid-Cell Transmisson Electron Microscopy. J. Am. Chem. Soc. 2019, 141, 10177–10182. 10.1021/jacs.9b04586. 31244172

[ref51] Patterson J. P.; Abellan P.; Denny M. S.; Park C.; Browning N. D.; Cohen S. M.; Evans J. E.; Gianneschi N. C. Observing the Growth of Metal-Organic Frameworks by in Situ Liquid Cell Transmission Electron Microscopy. J. Am. Chem. Soc. 2015, 137 (23), 7322–7328. 10.1021/jacs.5b00817. 26053504

[ref52] Hutzler A.; Fritsch B.; Jank M. P. M.; Branscheid R.; Martens R. C.; Spiecker E.; März M. In Situ Liquid Cell TEM Studies on Etching and Growth Mechanisms of Gold Nanoparticles at a Solid-Liquid-Gas Interface. Adv. Mater. Interfaces 2019, 6 (20), 1901027 10.1002/admi.201901027.

[ref53] Yamazaki T.; Kimura Y.; Vekilov P. G.; Furukawa E.; Shirai M.; Matsumoto H.; van Driessche A. E. S.; Tsukamoto K. Two Types of Amorphous Protein Particles Facilitate Crystal Nucleation. Proc. Natl. Acad. Sci. U. S. A. 2017, 114 (9), 2154–2159. 10.1073/pnas.1606948114. 28193873 PMC5338535

[ref54] Stawski T. M.; Roncal-Herrero T.; Fernandez-Martinez A.; Matamoros-Veloza A.; Kroger R.; Benning L. G. On Demand” Triggered Crystallization of CaCO 3 from Solute Precursor Species Stabilized by the Water-in-Oil Microemulsion. Phys. Chem. Chem. Phys. 2018, 20 (20), 13825–13835. 10.1039/C8CP00540K. 29745416

[ref55] Cookman J.; Hamilton V.; Price L. S.; Hall S. R.; Bangert U. Visualising Early-Stage Liquid Phase Organic Crystal Growth: Via Liquid Cell Electron Microscopy. Nanoscale 2020, 12 (7), 4636–4644. 10.1039/C9NR08126G. 32044911

[ref56] White E. R.; Mecklenburg M.; Shevitski B.; Singer S. B.; Regan B. C. Charged Nanoparticle Dynamics in Water Induced by Scanning Transmission Electron Microscopy. Langmuir 2012, 28 (8), 3695–3698. 10.1021/la2048486. 22320230 PMC3305795

[ref57] Powers A. S.; Liao H.-G.; Raja S. N.; Bronstein N. D.; Alivisatos A. P.; Zheng H. Tracking Nanoparticle Diffusion and Interaction during Self-Assembly in a Liquid Cell. Nano Lett. 2017, 17 (1), 15–20. 10.1021/acs.nanolett.6b02972. 27995796

[ref58] Jamali V.; Hargus C.; Ben-Moshe A.; Aghazadeh A.; Ha H. D.; Mandadapu K. K.; Alivisatos A. P. Anomalous Nanoparticle Surface Diffusion in LCTEM Is Revealed by Deep Learning-Assisted Analysis. Proc. Natl. Acad. Sci. U. S. A. 2021, 118 (10), 2017616118 10.1073/pnas.2017616118. PMC7958372 33658362

[ref59] Chen X.; Wen J. In Situ Wet-Cell TEM Observation of Gold Nanoparticle Motion in an Aqueous Solution. Nanoscale Res. Lett. 2012, 7, 1–6. 10.1186/1556-276X-7-598. 23107519 PMC3502471

[ref60] Touve M. A.; Carlini A. S.; Gianneschi N. C. Self-Assembling Peptides Imaged by Correlated Liquid Cell Transmission Electron Microscopy and MALDI-Imaging Mass Spectrometry. Nat. Commun. 2019, 10 (1), 1–12. 10.1038/s41467-019-12660-1. 31645558 PMC6811541

[ref61] Touve M. A.; Figg C. A.; Wright D. B.; Park C.; Cantlon J.; Sumerlin B. S.; Gianneschi N. C. Polymerization-Induced Self-Assembly of Micelles Observed by Liquid Cell Transmission Electron Microscopy. ACS Cent Sci. 2018, 4 (5), 543–547. 10.1021/acscentsci.8b00148. 29806000 PMC5968509

[ref62] Li C.; Tho C. C.; Galaktionova D.; Chen X.; Kral P.; Mirsaidov U. Dynamics of Amphiphilic Block Copolymers in an Aqueous Solution: Direct Imaging of Micelle Formation and Nanoparticle Encapsulation. Nanoscale 2019, 11, 2299 10.1039/C8NR08922A. 30662983

[ref63] Korpanty J.; Parent L. R.; Hampu N.; Weigand S.; Gianneschi N. C. Thermoresponsive Polymer Assemblies via Variable Temperature Liquid-Phase Transmission Electron Microscopy and Small Angle X-Ray Scattering. Nat. Commun. 2021, 12 (1), 1–8. 10.1038/s41467-021-26773-z. 34772926 PMC8589985

[ref64] Scheutz G. M.; Touve M. A.; Carlini A. S.; Garrison J. B.; Gnanasekaran K.; Sumerlin B. S.; Gianneschi N. C. Probing Thermoresponsive Polymerization-Induced Self-Assembly with Variable-Temperature Liquid-Cell Transmission Electron Microscopy. Matter 2021, 4, 722 10.1016/j.matt.2020.11.017.

[ref65] Rizvi A.; Patel U.; Ianiro A.; Hurst P. J.; Merham J. G.; Patterson J. P. Nonionic Block Copolymer Coacervates. Macromolecules 2020, 53 (14), 6078–6086. 10.1021/acs.macromol.0c00979.

[ref66] Rizvi A.; Mulvey J. T.; Patterson J. P. Observation of Liquid-Liquid-Phase Separation and Vesicle Spreading during Supported Bilayer Formation via Liquid-Phase Transmission Electron Microscopy. Nano Lett. 2021, 21 (24), 10325–10332. 10.1021/acs.nanolett.1c03556. 34890211

[ref67] Gibson W.; Patterson J. Observing Nano-Scale Dynamics of Active Soft Materials by In Situ Electrochemistry and Liquid Cell Transmission Electron Microscopy. Microscopy and Microanalysis 2022, 28 (S1), 96–99. 10.1017/S1431927622001295.35177139

[ref68] le Ferrand H.; Duchamp M.; Gabryelczyk B.; Cai H.; Miserez A. Time-Resolved Observations of Liquid-Liquid Phase Separation at the Nanoscale Using in Situ Liquid Transmission Electron Microscopy. J. Am. Chem. Soc. 2019, 141 (17), 7202–7210. 10.1021/jacs.9b03083. 30986043

[ref69] Schermelleh L.; Ferrand A.; Huser T.; Eggeling C.; Sauer M.; Biehlmaier O.; C Drummen G. P. Super-resolution microscopy demystified. Nat. Cell Biol. 2019, 21, 72 10.1038/s41556-018-0251-8. 30602772

[ref70] Ianiro A.; Wu H.; van Rijt M. M. J.; Vena M. P.; Keizer A. D. A.; Esteves A. C. C.; Tuinier R.; Friedrich H.; Sommerdijk N. A. J. M.; Patterson J. P. Liquid-Liquid Phase Separation during Amphiphilic Self-Assembly. Nature Chemistry 2019, 11 (4), 320–328. 10.1038/s41557-019-0210-4. 30778139

[ref71] Vratsanos M. A.; Gianneschi N. C. Direct Observation of Emulsion Morphology, Dynamics, and Demulsification. ACS Nano 2022, 16, 7783 10.1021/acsnano.2c00199. 35302741 PMC9836053

[ref72] Li M.; Yi L.; Sun C. Spontaneously Formed Multiscale Nano-Domains in Monophasic Region of Ternary Solution. J. Colloid Interface Sci. 2022, 628, 223–235. 10.1016/j.jcis.2022.07.152. 35932663

[ref73] Taylor P. Ostwald Ripening in Emulsions. Adv. Colloid Interface Sci. 1998, 75 (2), 107–163. 10.1016/S0001-8686(98)00035-9. 14672850

[ref74] Parent L. R.; Bakalis E.; Proetto M.; Li Y.; Park C.; Zerbetto F.; Gianneschi N. C. Tackling the Challenges of Dynamic Experiments Using Liquid-Cell Transmission Electron Microscopy. Acc. Chem. Res. 2018, 51 (1), 3–11. 10.1021/acs.accounts.7b00331. 29227618

[ref75] Bakalis E.; Parent L. R.; Vratsanos M.; Park C.; Gianneschi N. C.; Zerbetto F. Complex Nanoparticle Diffusional Motion in Liquid-Cell Transmission Electron Microscopy. J. Phys. Chem. C 2020, 124 (27), 14881–14890. 10.1021/acs.jpcc.0c03203. PMC8023318 33841603

[ref76] Iglicki D.; Goubault C.; Nour Mahamoud M.; Chevance S.; Gauffre F. Shedding Light on the Formation and Stability of Mesostructures in Ternary “Ouzo” Mixtures. J. Colloid Interface Sci. 2023, 633, 72–81. 10.1016/j.jcis.2022.11.060. 36436349

