# Supplementary material for: Ouzo Effect Examined at the Nanoscale via Direct Observation of Droplet Nucleation and Morphology
Source: ACS Cent Sci. 2023 Mar 8;9(3):457–65. doi: 10.1021/acscentsci.2c01194 (PMC10037490; doi:10.1021/acscentsci.2c01194)
Supplement: Supplementary file 1 — oc2c01194_si_001.pdf [file oc2c01194_si_001.pdf]

Ouzo Effect Examined at the Nanoscale via Direct Observation of Droplet Nucleation and Morphology

*Maria A. Vratsanos[a], Wangyang Xue[b], Nathan D. Rosenmann[a], Lauren D. Zarzar[b,c,d], and Nathan C. Gianneschi[a,e,f]**

[a] *Department of Materials Science & Engineering, Northwestern University, Evanston, Illinois 60208, United States.*

[b] *Department of Chemistry, The Pennsylvania State University, University Park, Pennsylvania 16802, United States*

[c] *Department of Materials Science and Engineering, The Pennsylvania State University, University Park, Pennsylvania 16802, United States*

[d] *Materials Research Institute, The Pennsylvania State University, University Park, Pennsylvania 16802, United States*

[e] *International Institute for Nanotechnology, Simpson Querrey Institute, Chemistry of Life Processes Institute, Northwestern University, Evanston, Illinois 60208, United States*

[f] *Department of Chemistry, Department of Biomedical Engineering, Department of Pharmacology, Northwestern University, Evanston, Illinois 60208, United States*

*Corresponding author email: nathan.gianneschi@northwestern.edu

Contents:

I. Materials
II. Sample Preparation
III. Liquid Cell Assembly
IV. Fluorescence and Optical Microscopy
V. Flow Analysis
VI. Microscope and Imaging Conditions
VII. Image Analysis
VIII. µFTIR Analysis
IX. Droplet Growth Data and Discussion
X. References

I. Materials

Trans-anethole (>98.0%) was purchased from TCI Tokyo, ethyl alcohol anhydrous (>99.98%) from (Electron Microscopy Science), and Nile Red from CHEM-IMPEX. All other reagents, including N,N-dimethylaniline, dioctyl sulfosuccinate, sodium salt (Aerosol OT/AOT), and 2,2,4-methylpentane (isooctane), were received from Sigma and used without further purification.

II. Sample Preparation

Solutions of *trans*-anethole in ethanol were prepared and stored for no more than 24 hours before use so as to ensure sample integrity. All formulations are given in volume percentages. To pre-form the droplets, an aliquot of this solution was chilled, and DI water was added dropwise until the solution was homogeneously cloudy. The same protocol was followed for the N, N-dimethylaniline solutions.

III. Liquid Cell Assembly

LPTEM experiments were performed using the Hummingbird Scientific Dual Flow Mixing holder as previously described.[1] Briefly, a non-glow discharged SiN_x chip was seated in the holder tip,

and an 0.8μL droplet of the sample was dropcast onto it. The top SiN_x chip was placed such that the rectangular windows were oriented orthogonally (**Figure S1**), and the top plate and clamp were used to seal the cell. The liquid cell's integrity was verified using an external pumping station fitted with an optical microscope, such that the windows could be visually inspected for fracture and to ensure they could withstand the vacuum of the microscope (8.6×10^{-6} mbar). The microfluidic lines were left unfilled but sealed during this test.

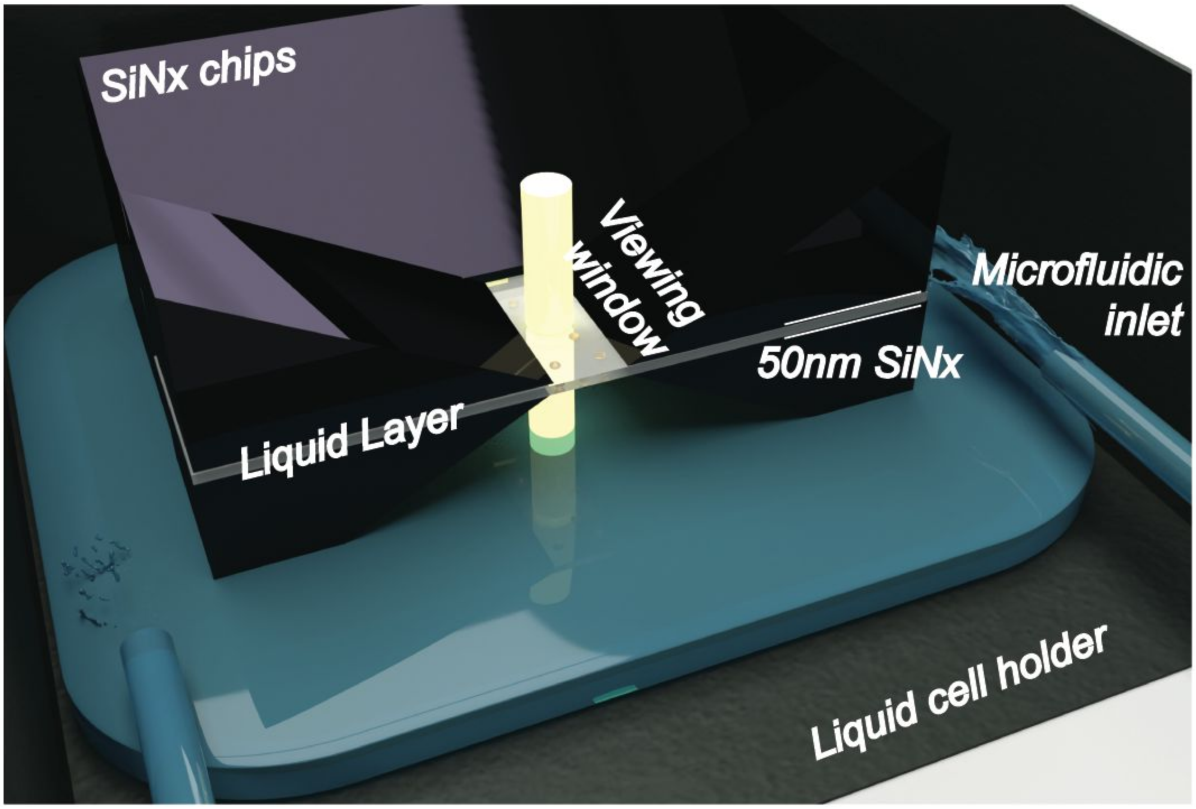

Figure S1 Schematic depiction of SiN_x liquid cell assembly. Notable features here include the 50 nm thick SiN_x windows through which the imaging occurs, the variable thickness liquid layer (ranging from 200-500 nm), and the microfluidic ports on the periphery of the SiN_x enclosure.

IV. Fluorescence and Optical Microscopy

Brightfield optical micrographs were taken on a Nikon Ti-U inverted microscope using an Imaging Source 23UX249 color camera. Nikon Plan Fluor 100x/1.30 Oil objective was used to image the samples. Differential interference contrast was used. Fluorescence images were taken by a Zeiss Axio Observer inverted microscope with Zen pro software and an Axiocam 503 mono camera. A Zeiss Plan-APOCHROMAT 63x/1.4 oil objective was used to observe the droplets and Nile Red, the fluorescent dye used for dying trans-anethole droplets, was excited by Colibri 7 LED light. A 91 HE CFP/YFP/mCherry filter was used to give off excitation from 494 – 528 nm and receive emission from 546 – 564 nm. The apparatus for containing the solution is as shown below (Figure S2). Adding 3 drops (~150 μL) of DI water to the 1 mL *trans*-anethole in ethanol solution would induce formation of *trans*-anethole droplets. To observe the droplets on an inverted microscope, 1 mL of *trans*-anethol / ethanol solution was transferred to a coverslip-bottom dish to which 3 drops of DI water is subsequently added.

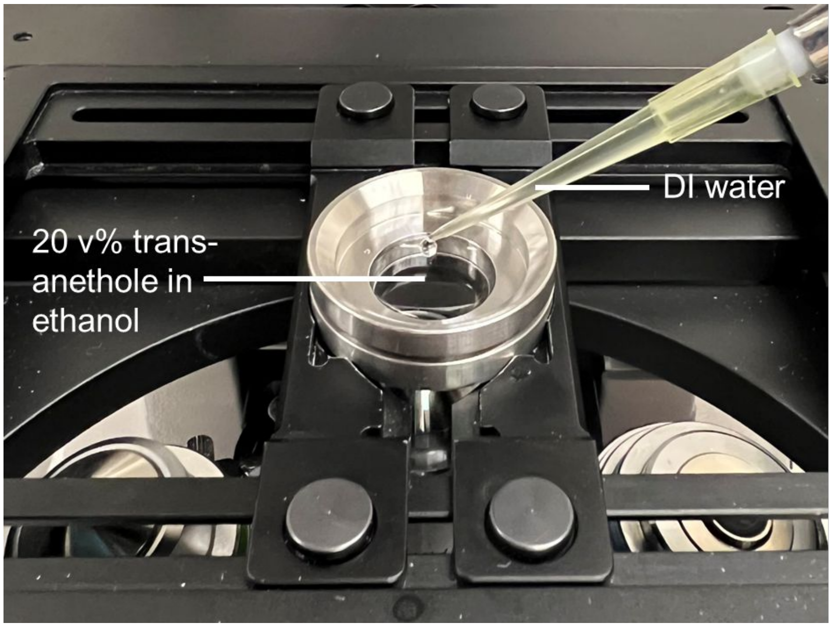

Figure S2 Experimental set up for optical and fluorescence microscopy. Sample chamber contains ethanol and trans-anethole, which is manually diluted via pipette.

V. Flow Analysis

The flow lines have an internal diameter of approximately 360 μm, and a length of approximately 40 cm. The fluid velocity varies linearly with volumetric flow rate, so under these constraints, so the fluid fronts were moving at approximately 0.49 mm/s and 0.16 mm/s at the higher and lower flow rates, respectively. Thus, diluent should have taken from 13-39 minutes to reach the tip of the holder. There is some variability associated with the actual mixing of the inlet solution and the sample between the chips. Because the solution is flowed into the chamber holding the SiN_x chips (Figure S1), there is some exterior volume that must be filled before mixing can occur.

Preliminary emulsification experiments were carried out with surfactant-loaded oil phases which were imaged prior to flowing in water. In such experiments, anisotropic appearance of the dispersed phase from the port of origin confirms these calculations and demonstrates the diffusive-driven nature of the *in situ* transport (**Figure S3**).

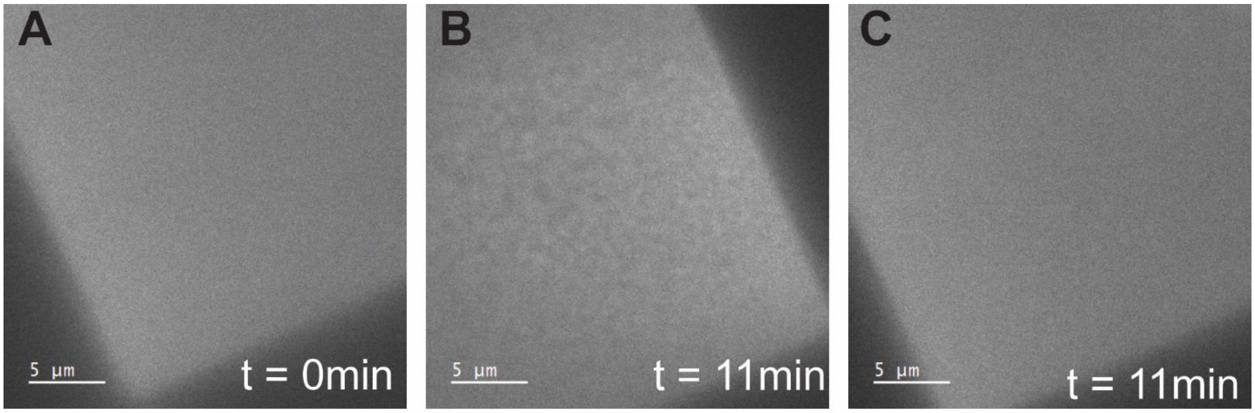

Figure S3 Time series of representative micrographs of i*n situ* experiment flowing water into sample of AOT dissolved in isooctane. (A) Initial image of cell prior to beginning flow of water (t=0 min), demonstrating homogeneous and structure-less morphology. Water flowed at 5 µl/min resulted in appearance of structures in one corner (B), but not another (C) after 11 minutes of dilution.

VI. Microscope and Imaging Conditions

A JEM-ARM300F (JEOL Ltd., Tokyo, Japan) transmission electron microscope operating at a voltage of 300keV and a current of 15μA was utilized to perform the *in situ* experiments. Images were acquired with a Gatan 2k x 2k OneView-IS CMOS camera (Gatan Inc., Pleasanton, CA,

USA) at an exposure of 0.5 s via Gatan Digital Micrograph imaging software (Roper Technologies, Sarasota, FL, USA). Electron fluxes were calculated using the measured beam current, which was calibrated previously with a Faraday Holder using the relevant apertures.

VII. Image Analysis

Image processing and analysis was done in ImageJ. Acquired images were rebinned to a dimension of 1024x1024 pixels and background adjusted by subtracting a Gaussian blurred duplicate. Videos were created by aligning and stacking images of the same field of view. Droplets were measured manually, as the contrast was insufficient to yield reliable automated thresholding.

VIII. μFTIR Analysis

In order to verify the molecular integrity of the *trans*-anethole after imaging, post-mortem μFTIR was performed. A Bruker MicroFTIR was used to analyze the windows of the SiN_x chips after the conclusion of the in *situ* experiment. The chips were removed from the holder, pried apart, and allowed to dry prior to analysis. Given the thickness of the SiN_x chips and windows, it was necessary to run these experiments in reflectance mode, which also required 500 scans in order to generate signal with sufficient intensity. 2cm wavelength resolution was used. As a control, the same *trans*-anethole solution was dropcast onto a SiNx chip and allowed to dry, so as to serve as

a representative spectrum of the material without imaging. High flux imaging conditions yielded visible damage in addition to spectra inconsistent with unimaged regions and controls, corroborating the low flux conditions used as safe for the material (Figure S4.

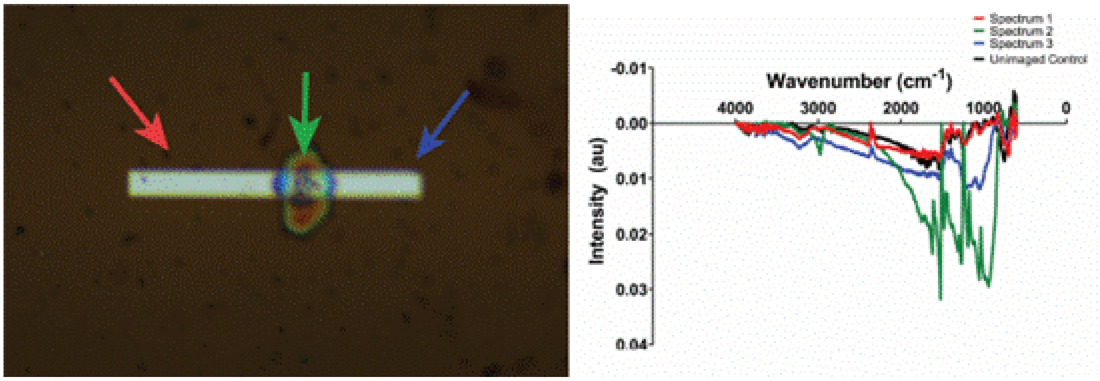

Figure S4 Time series of representative micrographs of i*n situ* experiment flowing water into sample of surfactant-containing oil. (A) Initial image of cell prior to beginning flow of water (t=0min), demonstrating homogeneous and structure-less morphology. Water flowed at 5 µl/min resulted in appearance of structures in one corner (B), but not another (C) after 11 minutes of dilution

IX. Droplet Growth Data and Discussion

Using the image analysis techniques outlined above, we analyzed droplet growth data from emulsification experiments across a range of concentrations (5, 10, and 20 v.% trans-anethole) and flow rates (1 or 3 µL/min) (**Figure S5**) in order to evaluate differences in nucleation and growth rates.

Growth rates were determined *via* logistic fit in Prism software, and k values (or logistic growth rates) are compared below (**Table S1**).

It was anticipated that slower flow rates would result in slower growth rates and a greater, more homogeneous droplet population than a higher flow rate, resulting from the increased equilibration time. A weak concentration dependence was observed, but the high standard deviations for these measurements rendered them statistically insignificant. However, one way ANOVA indicated significant differences in k values between flow rates (Figure S6, S7). The primary variation observed was droplet number as a function of *trans*-anethole concentration (Figure S66).

Table S 1 Tabulated droplet population and growth statistics for all experimental sample formulations

Content	Flow rate (μL/min)	Number of droplets	Growth constant (min^{-1})	Average starting diameter (μm)	Average final diameter (μm)
20 v% trans-anethole	3	49	0.2917±0.3325	1.39±0.26	1.97±0.50
20 v% trans-anethole	1	28	0.0240±0.0274	1.65±0.37	2.28±0.41
10 v% trans-anethole	3	15	0. 1293±0.2412	1.69±0.73	2.31±0.82
10 v% trans-anethole	1	6	0.04855 ± 0.02937	1.02±0.42	1.49±0.35
5 v% trans-anethole	3	3	0.05712±0.03628	1.69±0.38	2.96±0.45
20 v% dimethylaniline	3	>200	0.1824 ± 0.1353	1.33 ± 0.35	1.62 ± 0.44

Both linear and logarithmic growth fits were considered for both droplet diameter and the cube of the droplet radius (**Error! Reference source not found.**). Here, we see that the logistic fit has both a higher R^2 and lower standard error, indicating it is a better fit than the linear model. Additionally, we see that the r^3 plot is not well fit by a linear curve, which suggests that these droplet growth kinetics are not consistent with traditional models of Ostwald ripening

Table S 2 Comparison of curve fits for 5% trans-anethole data

	Linear Regression R^2	Linear Regression Error	Logistic Fit R^2	Logistic Fit Error
Droplet 1 - diameter	0.6413	0.2479	0.8858	0.1511
Droplet 2 - diameter	0.8962	0.1522	0.9024	0.1542
Droplet 3 - diameter	0.7602	0.2087	0.8102	0.1857
Droplet diameter - average	0.7659	0.203	0.866	0.1637
Droplet 1 – r^3	0.6673	0.3199	0.8198	0.2543
Droplet 2 – r^3	0.8694	0.5028	0.8687	0.5265
Droplet 3 – r^3	0.7672	0.4763	0.7637	0.5089
Droplet r^3 - average	0.7680	0.433	0.8174	0.4299

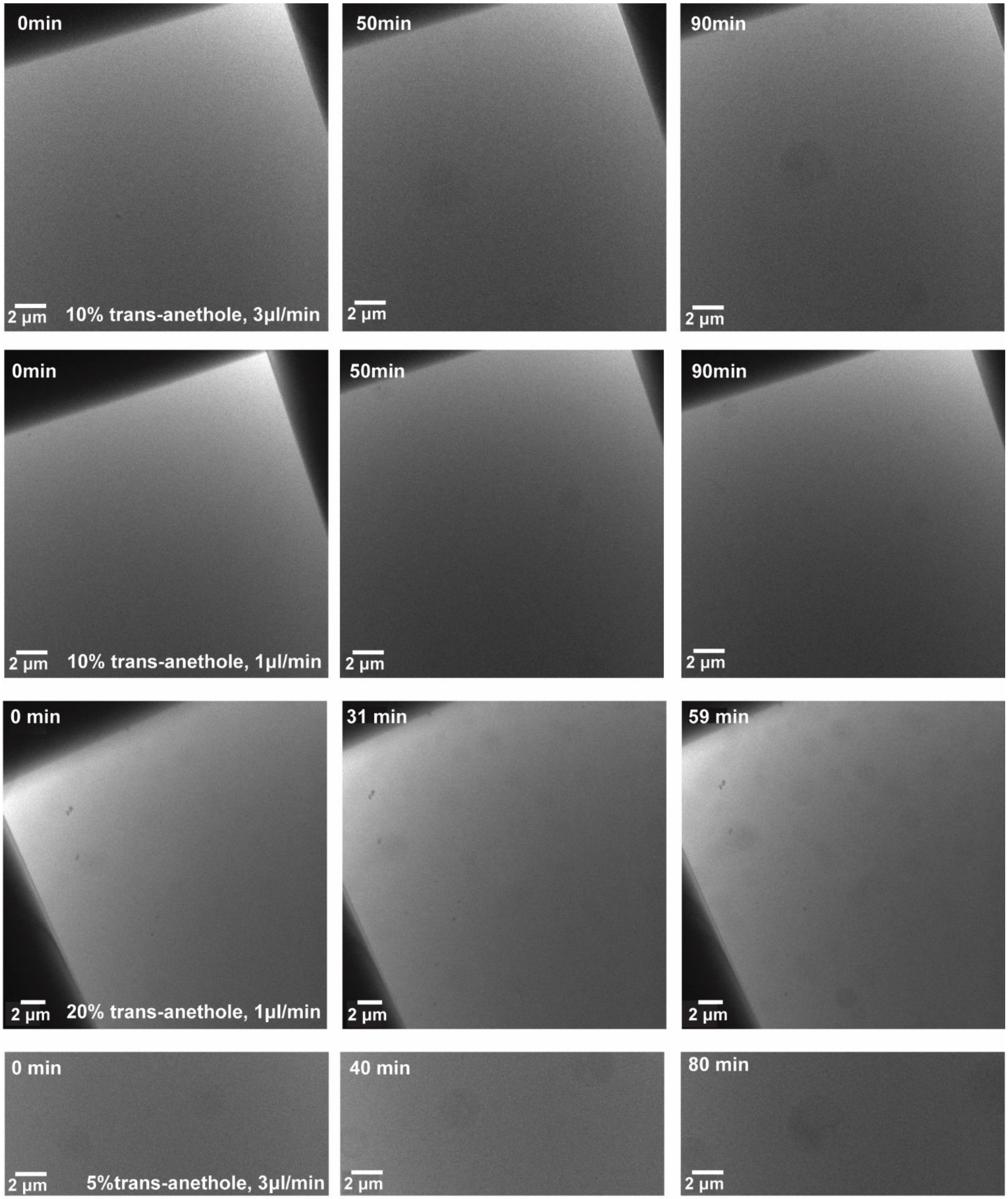

Figure S5 Representative time series of Ouzo nucleation across varying concentration and flow conditions. Times and scales as annotated.

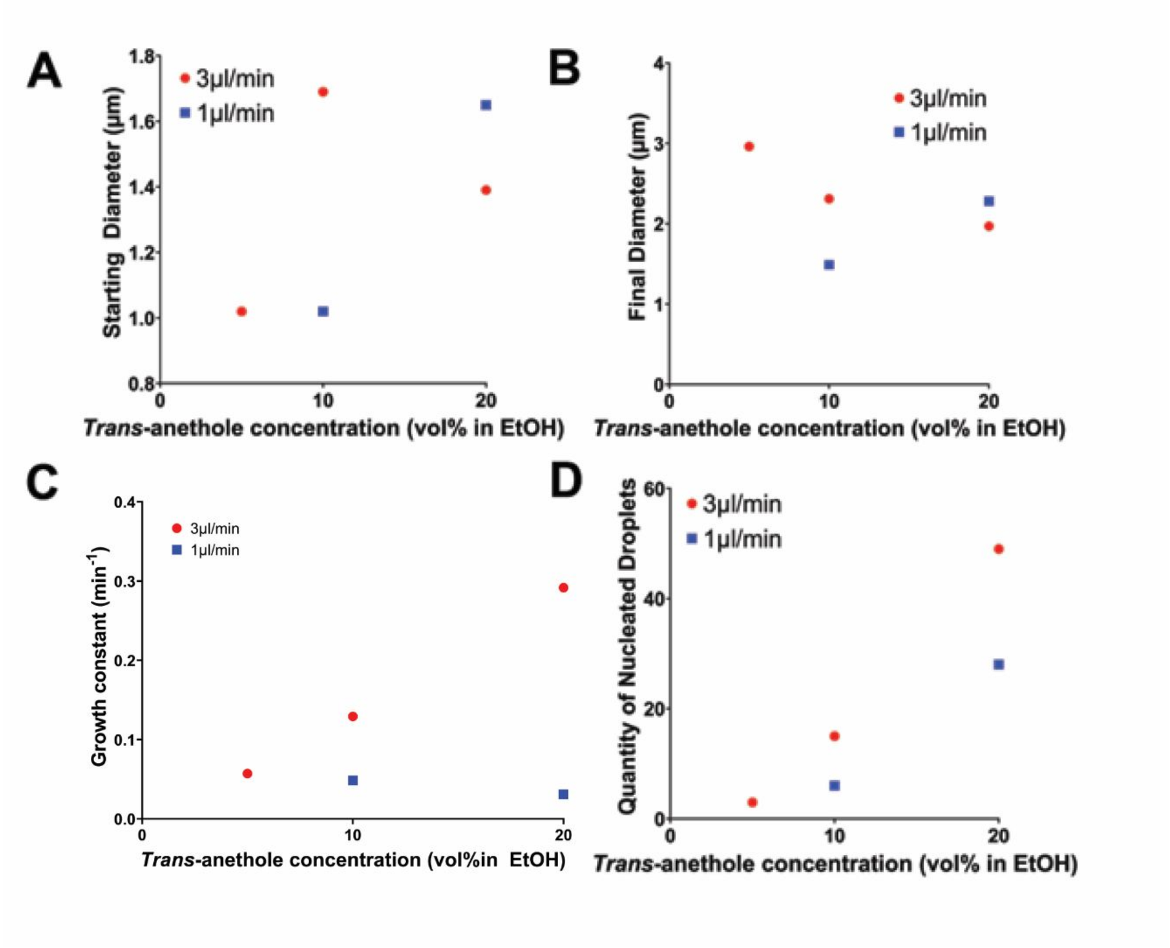

Figure S6 Particle growth statistics plotted as a function of trans-anethole concentration and dilution rate. (A) Droplet diameter at nucleation. (B) Droplet diameter at final imaging time point. (C) Growth rate in min^{-1} of droplets. (D) Number of droplets quantified.

One way ANOVA indicated that there is significant difference in growth constants as a function of flow rate ($p<0.001$) (**Figure S7**).

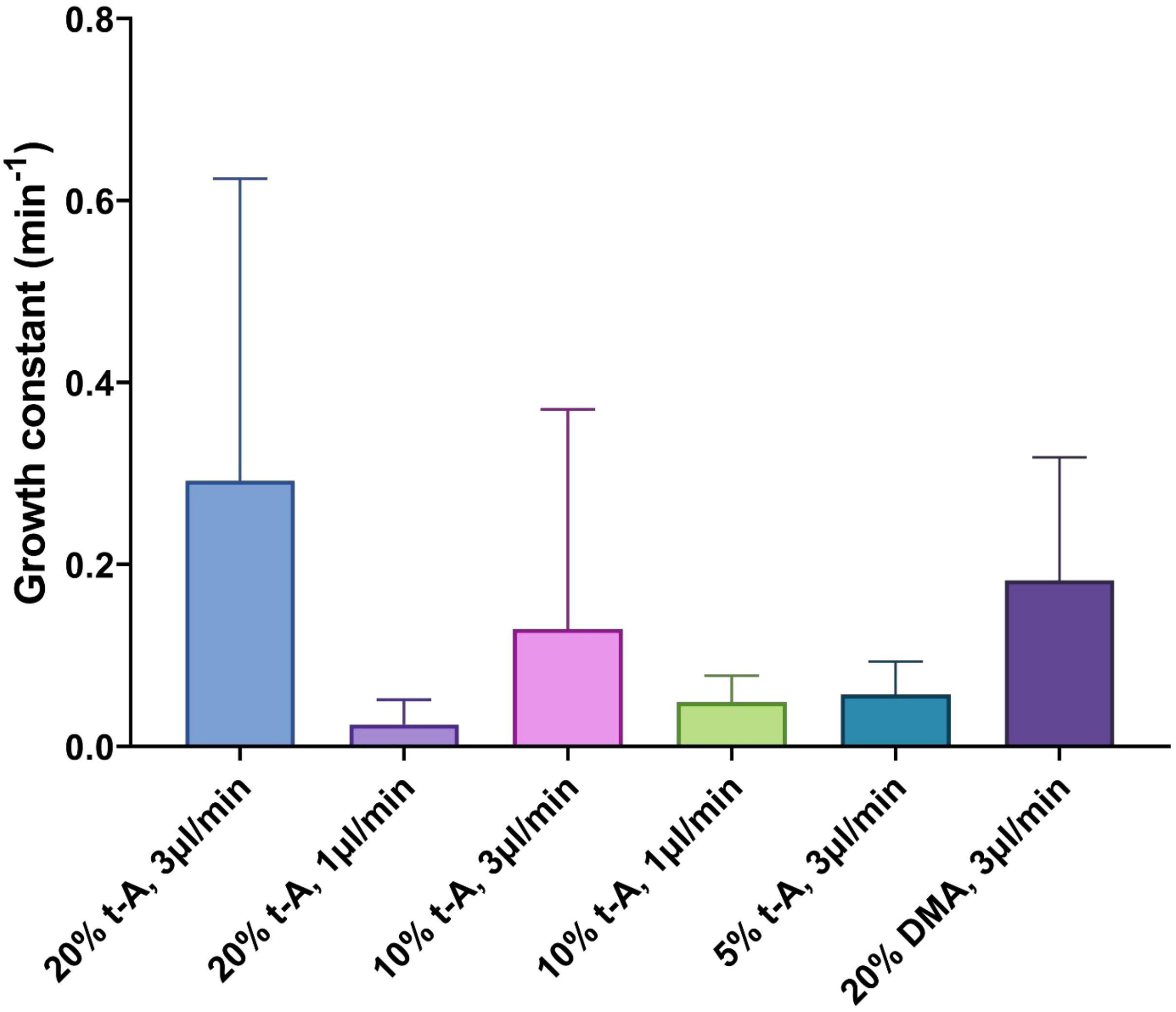

Figure S 7 Comparison of growth constants as a function of oil concentration and flow rate.

X. References

(1) Vratsanos, M. A.; Gianneschi, N. C. Direct Observation of Emulsion Morphology, Dynamics, and Demulsification. *ACS Nano* **2022**, acsnano.2c00199. https://doi.org/10.1021/ACSNANO.2C00199.
